# Systematic Exploration of Optimized Base Editing gRNA Design and Pleiotropic Effects with BExplorer

**DOI:** 10.1016/j.gpb.2022.06.005

**Published:** 2022-07-02

**Authors:** Gongchen Zhang, Chenyu Zhu, Xiaohan Chen, Jifang Yan, Dongyu Xue, Zixuan Wei, Guohui Chuai, Qi Liu

**Affiliations:** Translational Medical Center for Stem Cell Therapy and Institute for Regenerative Medicine, Shanghai East Hospital, Bioinformatics Department, School of Life Sciences and Technology, Tongji University, Shanghai 200092, China

**Keywords:** Gene editing, Base editing, Pleiotropy, CRISPR/Cas9, gRNA design

## Abstract

**Base editing** technology is being increasingly applied in genome engineering, but the current strategy for designing guide RNAs (gRNAs) relies substantially on empirical experience rather than a dependable and efficient *in silico* design. Furthermore, the pleiotropic effect of base editing on disease treatment remains unexplored, which prevents its further clinical usage. Here, we presented BExplorer, an integrated and comprehensive computational pipeline to optimize the design of gRNAs for 26 existing types of base editors *in silico*. Using BExplorer, we described its results for two types of mainstream base editors, BE3 and ABE7.10, and evaluated the pleiotropic effects of the corresponding base editing loci. BExplorer revealed 524 and 900 editable pathogenic single nucleotide polymorphism (SNP) loci in the human genome together with the selected optimized gRNAs for BE3 and ABE7.10, respectively. In addition, the impact of 707 edited pathogenic SNP loci following base editing on 131 diseases was systematically explored by revealing their pleiotropic effects, indicating that base editing should be carefully utilized given the potential pleiotropic effects. Collectively, the systematic exploration of optimized base editing **gRNA design** and the corresponding pleiotropic effects with BExplorer provides a computational basis for applying base editing in disease treatment.

## Introduction

Base editing technology has shown great potential in gene engineering due to its high efficiency, lack of need for donor DNA, and independence from DNA double-strand breaks. It could directly convert one base or base pair into another, and thus has been successfully applied in diverse species for genome engineering. It has also demonstrated great capacity in clinical disease treatment [1]. In 2016, Komor et al. proposed a base editor based on CRISPR/Cas9, which includes three main components, guide RNA (gRNA), Cas9 protein, and cytosine deaminase, and achieved precise replacement for single base [2]. Recently, many studies on base editors have been published, such as BE3, BE-PLUS, ABE7.10, and SaKKH-ABE, most of which were derived from the CRISPR/Cas9 system [2], [3], [4], [5]. Previous studies have demonstrated that the choice of gRNAs is an important factor affecting the actual editing effect of CRISPR/Cas9 [6]. In base editors, gRNAs also play an important role, guiding the fusion protein to bind to a specific site similar to that of CRISPR/Cas9. Therefore, there are substantial similarities in their gRNA structures and the optimized design rules of gRNA. Nevertheless, the activity window of the base editors and the protospacer adjacent motif (PAM) diversity require that different gRNA design strategies should be presented for base editing from those of CRISPR/Cas9. Limited tools have been developed for designing gRNAs for several kinds of base editors, and they can be divided into two categories which are machine learning-based and scoring function-based according to the used algorithm. BE-Hive, DeepBaseEditor, and CGBE-Hive [7], [8], [9] can be classified as machine learning-based tools. These tools establish several machine learning models to predict base editing results, but their prediction results are difficult to explain clearly in specific biological criteria. Scoring function-based tools have better performance in biological criterion explaining such as BEable-GPS, *beditor*, and BE-FF [10], [11], [12]. BEable-GPS establishes a gRNA design and searching system for base editors by taking PAM and the activity window as the main constraints. *beditor* integrates certain evaluation criteria including the PAM, the activity window, and off-target assessment. The evaluation criteria selected by BE-FF are the PAM, the activity window, and off-target assessment limited to NGG-based base editors. However, the recommendation of the best gRNA can be improved by a comprehensive consideration of existing factors that may influence base editing efficiency, such as activity window, GC content, SNP, off-target effects, and continuous identical bases.

Another important issue in the application of base editing is the evaluation of the pleiotropic effect. Pleiotropic effect is the effect of a single genetic locus on multiple phenotypes that may seem unrelated [13]. Genes that can affect the expression of multiple phenotypes are referred to as pleiotropic genes, and mutations in these genes may affect several phenotypes at the same time [14]. Previous studies have shown that many genes related to human diseases are also pleiotropic genes, such as *HTT* and *Hbb*. Mutation of the *HTT* gene can cause Huntington’s disease, while patients can show a notable increase in fecundity and experience lower rates of cancer [15], [16]. Mutations in the *Hbb* gene cause sickle cell anemia, but they have also been reported to improve individual survival in tropical regions via increased resistance to malaria [17]. In clinical applications, the impact of changes in the target gene on other disease phenotypes should be carefully considered in base editing to avoid changes in unexpected phenotypes. However, the pleiotropic effect in base editing has not been systematically explored.

In this study, we presented BExplorer, an integrated and comprehensive computational pipeline for optimally designing gRNA *in silico* for 26 existing types of base editor and evaluating the pleiotropic effects of the corresponding base editing loci. BExplorer was applied to two types of mainstream base editor, BE3 and ABE7.10, and 524 and 900 editable pathogenic SNP loci in the human genome together with the selected optimal gRNAs for these two types of base editors were obtained, respectively. In addition, we systematically explored the impact of the edited SNPs on various unexpected diseases in the application of base editors by revealing the pleiotropic effects of 707 pathogenic SNP loci on 131 diseases. Our comprehensive analyses indicate that (1) existing base editors have great potential to treat a variety of human diseases caused by point mutations. Many disease-related mutant base sites have multiple feasible gRNAs that can be selected with different pleiotropic risks; therefore, it is necessary to evaluate and screen the predicted gRNAs by carefully designing an optimal gRNA selection strategy. (2) Pleiotropy is universal among human pathogenic SNPs. It is necessary to consider the impact on other diseases caused by pleiotropy when applying base editing to correct pathogenic SNPs for disease treatments in clinic. By applying BExplorer, for the first time, we obtained a comprehensive set of editable pathogenic SNPs with weak pleiotropic effects as a candidate set, serving as an appropriate base editing locus resource for potential gene therapies and related clinical utilities with base editing.

## Implementation

### Editable base editing site screening strategy

Given the characteristics of base editing, we selected a batch of features and criteria that can be used for base editing site screening. The five screening criteria were target site base type, PAM sequence, activity window, the number of continuous identical bases, and the proportion of GC bases.

#### Target site base type matching

BExplorer first checks whether the target base matches the selected base editor. If it does not, “base_match_error” is output. The user version of BExplorer regards the base at the target site as C [in which case the selected base editor is cytosine base editor (CBE)] or A [in which case the selected base editor is adenine base editor (ABE)].

#### Downstream PAM checking

After performing the previous step, BExplorer searches for a PAM sequence in the downstream sequence over a specific length. If a PAM sequence is found within the specified length, the site is retained; otherwise, it is considered as a failed site and is marked with “no_PAM”.

#### Activity window screening

This step is to screen the target site based on the activity window. If the target site is located in the activity window, it is retained; otherwise, the site is considered as a failed site and is marked with “activity_window_error”.

#### Number of continuous identical bases

Previous studies have shown that less than seven continuous identical bases are more likely to increase the binding efficiency of gRNA [18]. Therefore, following the previous screening step, the primary candidate gRNAs are obtained. Candidate gRNAs with < 7 continuous identical bases is retained; if all candidate gRNAs have ≥ 7 continuous identical bases [19], the site is considered as a failed site and is marked with “continuous_identical_base_error”.

#### Proportion of GC bases

Previous studies have demonstrated that gRNA sequences with very high or low GC content are less effective against their targets [20]. Several studies have indicated that in order to obtain effective gRNAs, with 30% as a minimum threshold, the maximum threshold between 70%–80% is more reasonable GC content range [20], [21]. Therefore, we chosen 30%–75% as the GC content criterion to obtain candidate gRNAs. If there are candidate gRNAs with 30% ≤ GC content ≤ 75%, the site is retained; if the GC content of all candidate gRNAs does not meet this criterion, the site is considered as a failed site and is marked with “GC_ratio_error”.

### Ranking and evaluating the candidate gRNAs

The base editing system is based on the CRISPR/Cas9 system, and both have the same characteristics in many aspects. We incorporated several features of the CRISPR/Cas9 system to design the scoring and sorting criteria that can be used for evaluating base editing candidate gRNAs. The five evaluation criteria include the number of bases identical to the target base in the activity window, the GC content in the gRNA sequence, the number of repeated gRNA sequences in the whole genome, the number of SNPs in the gRNA sequence, and the potential off-target effects.

#### Number of bases identical to the target base in the activity window

The base editor converts all bases identical to the target base in the activity window. The fewer bases in the activity window that are the same as the target site, the higher the efficiency of gene editing will be [2] and, therefore, the higher the candidate gRNAs will be ranked.

#### Proportion of GC bases

Previous studies have shown that higher GC content gives more stability to RNA–DNA hybrid [22]. Therefore, higher GC content is more likely to induce gRNA off target due to its more tolerance to mismatches. The lower GC content of the candidate gRNA is, the higher ranking the candidate gRNA has.

#### Number of repeated gRNA sequences in the whole genome

The fewer repeats of candidate gRNA sequences there are in the genome, the higher the candidate gRNAs will be ranked.

#### Number of SNPs in the gRNA sequence

By using VCFtools [23], we obtained the number of human reference SNPs for each candidate gRNA sequence collected from the dbSNP database. The fewer SNPs there are in the sequence, the higher the candidate gRNA will be ranked.

#### Potential off-target effects

We applied the off-target effect prediction tool Cas-OFFinder [24] to predict off-target effects of each candidate gRNA and obtain their potential off-target sites. The CFD algorithm is applied to score the off-target probability for each candidate gRNA. The higher the CFD score is, the higher the probability of off-target effects is. Finally, all the CFD scores are averaged as one off-target effect score for the candidate gRNA. The lower the off-target effect score of the candidate gRNA is, the higher ranking the candidate gRNA has.

#### Ranking aggregation

We used the robust rank aggregation (RRA) algorithm to integrate all rankings from five perspectives [25]. RRA is a widely used rank aggregation algorithm that can combine preference lists from different perspectives into a single ranking in case of certain influence values of each perspective are unknown and obtain results that are robust to noise. Finally, we obtained the RRA score of each candidate gRNA. The smaller the RRA score is, the higher priority the candidate gRNA has to be selected for the target site.

### Evaluation of the pleiotropic effect by exploring human pathogenic SNPs in base editing

A human pathogenic SNP dataset containing 14,546 human pathogenic SNPs and corresponding phenotype data was generated by curating all the human SNP phenotype data marked with “pathogenic” from the Online Mendelian Inheritance in Man (OMIM) database [26]. The detailed procedure is listed: first, 131 phenotypes of human diseases with more than 1000 cases were screened from the UK Biobank, which contains a genome-wide association study (GWAS) on 361,194 participants, and 4203 human phenotypes together with 13,791,468 SNP mutation sites were collected [27]. By integrating the GWAS data from Biobank, we obtained a GWAS dataset containing 131 human diseases. Then, we chose the allelic substitution effect coefficient *β* as the correlation fitting coefficient to reveal the promotion and inhibition effects of SNPs on the phenotypes. The *P* value was calculated to indicate the strength of the association between SNPs and phenotypes.

To reliably evaluate the correlation between SNPs and phenotypes, we established a fitting coefficient conversion model according to the formula proposed by Pirinen and his colleagues [28].

(1) $$\beta =\beta_{\mathrm{obs}}{\left(\phi \left(1-\phi \right)+0.5\left(1-2\phi \right)\left(1-2\theta \right)\beta_{\mathrm{obs}}-\frac{0.084+0.9\phi \left(1-2\phi \right)\theta \left(1-\theta \right)}{\phi \left(1-\phi \right)}\beta_{\mathrm{obs}}^{2}\right)}^{-1}$$

where *β* represents allelic substitution effect, *β*_*obs*_ represents allelic substitution effect on the observed scale, *Φ* represents proportion of disease cases in the data, and *θ* represents reference allele frequency in the data.

Subsequently, we compared the human disease SNP dataset with the integrated human disease GWAS dataset and identified 707 human pathogenic single-base mutations in both. Using the pleiotropy evaluation model, we calculated the allelic substitution effect coefficient for each SNP with the alleles of 131 human diseases. Finally, we integrated all the data to obtain the results from the pleiotropy analysis of human pathogenic SNPs.

## Results

### General framework of BExplorer

**Figure 1:**
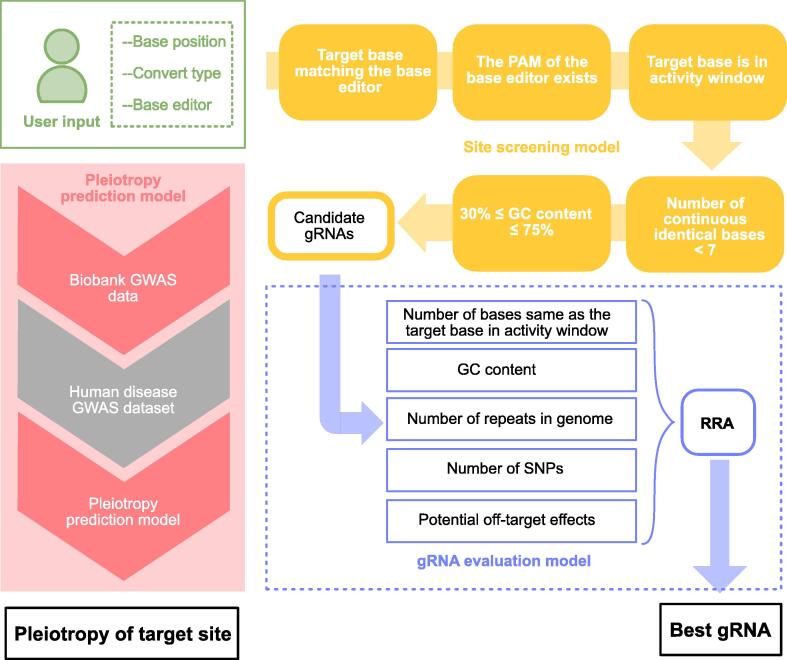
**The computational framework of BExplorer** BExplorer consists of three parts: site screening model, gRNA evaluation model, and pleiotropy prediction model. After the user enters the information (green part), the site screening model (yellow part) judges whether the target site is editable by the desired base editor according to five screening criteria. The gRNA evaluation model (blue part) evaluates each candidate gRNA and ranks the best gRNA by integrating five kinds of features. Pleiotropy prediction model (red part) predicts the pleiotropy of the target site and demonstrates the corresponding pleiotropy risk in base editing. PAM, protospacer adjacent motif; SNP, single nucleotide polymorphisin; RRA, robust rank aggregation; GWAS, genome-wide association study.

The general computational framework of BExplorer consists of the following steps (Figure 1): (1) BExplorer obtains user input information, including the target base location, the expected base conversion type, and the expected base editor. (2) The *in silico* screening model in BExplorer analyzes the input information to determine whether the expected site can be edited by the expected base editor. The *in silico* screening strategy for gRNA design performs the following five checks: (i) whether the target base matches the selected base editor, (ii) whether the PAM sequence is available over a certain sequence length downstream, (iii) whether the target base is in the activity window, (iv) whether the maximum number of continuous identical bases is less than 7, and (v) whether the percentage of GC bases is between 30% and 75%. (3) All valid gRNAs that pass the BExplorer screening model are presented. Next, the evaluation model in BExplorer scores all the candidate gRNAs to identify the best one. The evaluation model evaluates candidate gRNAs based on the following five criteria: (i) the number of bases identical to the target base in the activity window, (ii) the proportion of GC bases in the candidate gRNA, (iii) the number of repeat gRNAs in the whole genome, (iv) the number of SNPs, and (v) the potential off-target effects. Finally, we obtain the best gRNA for the target site using a RRA method to rank all the candidate gRNAs by the five scores. (4) BExplorer provides a pleiotropic prediction model to predict the impact of site changes corresponding to certain pathogenic SNPs on a comprehensive GWAS dataset of 131 diseases.

### Applying BExplorer to exploring human pathogenic SNPs in base editing

**Figure 2:**
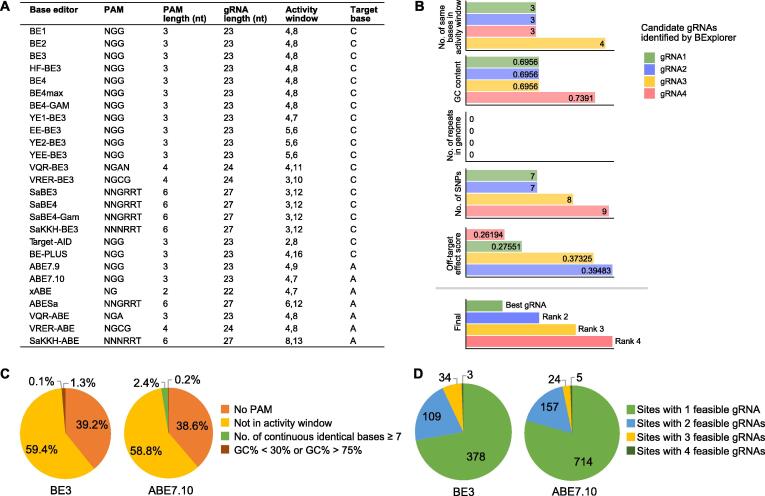
**Applying BExplorer to the analysis of human pathogenic SNP dataset** **A.** Twenty-six base editors with various features can be selected in BExplorer, including CBE and ABE. **B.** Evaluating each candidate gRNA based on five features and identifying the best gRNA to precisely edit the target site. Taking Chr2:26495075 as an example, BExplorer identified four candidate gRNAs (green, blue, yellow, and red) and evaluated them based on five features (above the gray line), and finally identified the best gRNA by integrating all the ranking lists (below the gray line). **C.** Distribution of the non-editable pathogenic SNPs in the case of target site matching base editor. **D.** Distribution of the number of editable sites with different numbers of feasible gRNAs in human pathogenic SNPs. CBE,  cytosine base editor; ABE, adenine base editor.

To investigate the potential applications of base editing in human point mutation disease treatment, we applied BExplorer to the human pathogenic SNP dataset. Twenty-six common base editors with different characteristics, such as target base, PAM sequence, and gRNA length, were integrated into BExplorer [2], [3], [4], [5], [29], [30], [31], [32], [33], [34], [35], [36], [37] (Figure 2A), and different gRNA prediction and evaluation strategies were output by BExplorer. Among the evaluated base editors, BE3 and ABE7.10 are currently widely used as a CBE and ABE, respectively. We screened 14,546 human pathogenic SNPs and corresponding phenotype data from the OMIM database [26] and integrated them into a human pathogenic SNP dataset. The evaluation models of gRNA affinity in BE3 and ABE7.10 were established separately by changing certain parameters in BExplorer and they were applied to the human pathogenic SNP dataset. After screening, we obtained 524 pathogenic SNP sites that are expected to be corrected by BE3 (Table S1) and 900 pathogenic single-base mutation sites that are expected to be corrected by ABE7.10 (Table S2). The results indicate that there are a large number of pathogenic SNPs that can be corrected by these base editors, suggesting that base editing has great potential in the treatment of human point mutation diseases. Since one site may correspond to multiple feasible gRNAs, we applied BExplorer to evaluate each candidate gRNA based on the abovementioned five criteria and combined all the rankings to finally obtain the best gRNA for precise editing of the target site (Figure 2B).

To study why a number of pathogenic SNPs could not be edited by the base editor, we investigated the sites that did not pass BExplorer site screening (Figure 2C). The results indicated that the main reasons that the pathogenic SNPs did not pass the screening were that the PAM sequence was absent downstream and the target site was not in the activity window. These sites that failed accounted for 98.6% and 97.4% of the sites screened for BE3 and ABE7.10, respectively. Next, we investigated the importance of gRNA affinity prediction and evaluation in base editing. We applied BExplorer to predict all feasible gRNAs for each editable site and finally obtained 710 feasible gRNAs for 524 BE3-editable sites and 1120 feasible gRNAs for 900 ABE7.10-editable sites, respectively. Our analyses indicated that BE3 and ABE7.10 had similar distributions of viable gRNAs for their respective editable sites (Figure 2D). Among the editable sites for BE3, sites with one (*n* = 378) or two (*n* = 109) feasible gRNAs accounted for 72.14% and 20.80% of the total number of editable sites, respectively, while the numbers of sites with three (*n* = 34) or four (*n* = 3) feasible gRNAs were relatively small, accounting for only 6.49% and 0.57% of the total sites, respectively. Similarly, among the editable sites for ABE7.10, sites with one (*n* = 714) or two (*n* = 157) feasible gRNAs accounted for 79.33% and 17.44% of the total number of editable sites, respectively, while the numbers of sites with three (*n* = 24) or four (*n* = 5) feasible gRNAs were relatively small, accounting for only 2.67% and 0.56% of the total sites, respectively. Unlike the gRNA design of the CRISPR/Cas9 gene editing system, base editing is limited by the activity window, and consequently, the number of feasible gRNAs is relatively small. Consistent with a previous study showing that ABE7.10 has a smaller activity window [1], our results indicate that the average number of feasible gRNAs per site for ABE7.10 is less than that for BE3, further indicating that the smaller activity window is accompanied by a lower average number of feasible gRNAs per site for base editors. Moreover, one base editing site is often accompanied by multiple feasible gRNAs, indicating that the evaluation of candidate gRNAs for optimized gRNA selection is a necessary step for base editing.

### Evaluation of the pleiotropic effect by exploring human pathogenic SNPs in base editing

**Figure 3:**
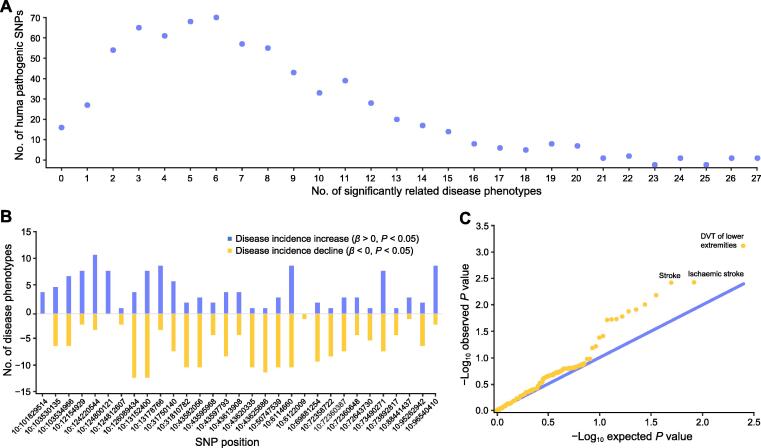
**Pleiotropy prediction for human pathogenic SNPs** **A.** Pleiotropy strength of human pathogenic SNPs. Most pathogenic SNPs are significantly (*P* < 0.05) associated with disease phenotypes within 10. **B.** Pleiotroic promotion effect (*β* > 0, *P* < 0.05) and inhibition effect (*β* < 0, *P* < 0.05) on chromosome 10 pathogenic SNPs. In most pathogenic SNPs, the pleiotropy promotion and inhibition effects exist simultaneously, and the pleiotropy strength varies from one SNP to another. **C.** Normality test on one SNP (example site: Chr1:26136244). The observed *P* value is consistent with the expected *P* value in weakly correlated disease phenotypes, while in significantly correlated disease phenotypes including DVT of lower extremities and ischemic stroke, the observed *P* value exceeds the expected *P* value. DVT, deep vein thrombosis.

To explore the impact of human pathogenic SNP pleiotropy on base editing in disease treatment, we applied BExplorer to predict the pleiotropic effects of SNPs in the human pathogenic SNP dataset. A total of 131 human disease phenotypes with more than 1000 cases from the UK Biobank database [27] were selected, and their GWAS data were used for pleiotropy prediction. We applied the pleiotropy prediction model to the human pathogenic SNP dataset and analyzed the pleiotropy of 707 human pathogenic SNPs. First, we analyzed the pleiotropy strength of the human pathogenic SNPs (Figure 3A). The results showed that most human pathogenic SNPs were significantly associated with more than one disease phenotype (*P* < 0.05). A total of 577 SNPs were significantly associated with fewer than 10 disease phenotypes, accounting for 81.6% of the total number of SNPs; 121 SNPs were significantly associated with 10–20 disease phenotypes, accounting for 17.1% of the total number of SNPs; and only 9 SNPs were significantly associated with more than 20 disease phenotypes, accounting for only 1.3% of the total SNPs. It is worth noting that the Chr1:12069698 SNP was associated with 27 disease phenotypes, and editing of this SNP would trigger a large number of unexpected disease phenotype changes. Our analysis shows that pleiotropy is universal among human pathogenic SNPs, and most pathogenic SNPs are significantly associated with fewer than 10 disease phenotypes.

Next, we explored the pleiotropic promotion and inhibition effects from the human pathogenic SNPs. A promotion effect indicates that the pathogenic SNP promotes the occurrence of diseases, and an inhibition effect means that the pathogenic SNP inhibits the occurrence of diseases. We analyzed the significant pleiotropic promotion (*β* > 0, *P* < 0.05) and inhibition (*β* < 0, *P* < 0.05) effects of all the pathogenic SNPs and selected pathogenic SNPs on chromosome 10 as an example in Figure 3B. The results showed that for most pathogenic SNPs, pleiotropic promotion and inhibition effects existed simultaneously, and the pleiotropy strength varied from one SNP to another. For instance, the Chr10:13152400 SNP significantly promotes the occurrence of 8 diseases and significantly inhibits the occurrence of 12 diseases, therefore, this SNP is not suitable for base editing studies on individual disease phenotypes. The Chr10:6122009 SNP, on the other hand, only significantly inhibits one disease and significantly promotes none. Therefore, this SNP is a more appropriate choice for base editing studies on individual disease phenotypes.

Third, we analyzed the genes *TP53* and *HBG1*, which are commonly used in base editing research to study the effect of pleiotropy as a demonstration example. *TP53* is an important tumor suppressor gene, and its point mutations increase the risk of human cancer. Previous studies have demonstrated that base editing has great application potential in correcting *TP53* point mutations [2]. For this study, we conducted pleiotropy analysis on common SNPs of *TP53*, and the results showed that the pleiotropic effect of *TP53* was relatively strong. rs78378222 is a common SNP of *TP53*, and it significantly increases (*β* > 0, *P* < 0.05) the incidence of 17 phenotypes, including gastroduodenal ulcers, malignant neoplasms of the prostate, and meniscus derangement, and significantly reduces (*β* < 0, *P* < 0.05) the incidence of 3 phenotypes, including disorders of the lens and actinic keratosis. The second gene is *HBG1*, which is a common hemoglobin gene prevalently used in base editing research [3]. We analyzed the pleiotropic effect of the common SNP rs1061234 of *HBG1*, and the results showed that this SNP significantly increased the incidence of 7 phenotypes, including venous thromboembolism, coronary atherosclerosis, and deep vein thrombosis (DVT) of the lower extremities; however, no significant decrease in the incidence of other phenotypes was detected, demonstrating that the pleiotropic effect of *HBG1* is relatively weak. Collectively, our analysis indicated that pleiotropic analysis of the target gene in base editing applications is necessary, but has been ignored in previous studies.

To facilitate the usage of BExplorer for the base editing community, we screened 111 pathogenic SNPs with weak pleiotropic effects that can be used as appropriate and safe base editing SNPs, providing a resource for potential gene therapies and biomedical studies (Table S3). To assess the pleiotropy prediction for one SNP, we performed a normality test on the Chr1:26136244 site (Figure 3C). The analysis revealed an observed *P* value consistent with the expected *P* value in weakly correlated diseases. However, for significantly correlated disease phenotypes, including DVT of the lower extremities and ischemic stroke, the observed *P* value exceeded the expected *P* value, demonstrating that the proposed pleiotropic prediction model is reliable.

## Discussion

The current base editing gRNA design relies substantially on personal experience rather than reliable and efficient computational design. In this work, we presented BExplorer, an integrated computational pipeline for computationally gRNA design for 26 base editors, and evaluated their pleiotropic effects. In addition, the evaluation of pleiotropic effect by editing target sites with base editors has great potential to contribute to a more complete understanding of the risks of base editing in clinic. In summary, our comprehensive analysis indicates that BExplorer is an efficient computational tool for identifying editable sites, distinguishing the best gRNA from multiple feasible gRNAs, and evaluating the impact of target sites on unknown diseases. We validated the necessity and reliability of BExplorer and investigated its potential applications in treating human point mutation diseases and risk evaluation. Our analysis indicates that the restrictions of the PAM sequence and activity window are two main reasons that certain target sites failed to be edited, providing a direction for further optimization of the base editor. In addition, the pleiotropic assessment shows that pleiotropy is universally present in human pathogenic SNPs, and most pathogenic SNPs are significantly associated with fewer than 10 diseases. By analyzing the pleiotropy promotion and inhibition effects for all pathogenic SNPs, we found that these two effects exist simultaneously for most SNPs, and the strength of pleiotropy at different sites is quite different. These results further indicate that the pleiotropy of SNPs will be an important factor affecting the application of base editing. In summary, BExplorer not only provides a reliable gRNA design strategy to improve the efficiency of base editing but also presents a reliable way to evaluate the pleiotropy of target sites and reduce the risk of applying base editing in the clinic.

Our current study is limited by the human pathogenic SNP dataset, and the prediction of RNA-level off-targets induced by base editors is unexplored in the current version of BExplorer due to its complexity and diversity. Additionally, the pleiotropy effect will be influenced by unexpected base editing if there are multiple editable bases within the activity window. However, the influence tends to be weak because the target base and unexpected editable bases within the activity window are likely to be in the same gene. Finally, our findings are limited by the abundance of human disease phenotype GWAS data. Future development of BExplorer will include three main updates: (1) more reasonable screening and evaluation criteria for improving the design of base editing gRNAs; (2) more types of base editors into the pipeline for further increasing the number of editable sites; and (3) more human disease GWAS data for expanding the evaluation of pleiotropy.

## Code availability

BExplorer is freely available at https://github.com/bm2-lab/BExplorer.

## Competing interests

The authors have declared no competing interests.

## CRediT authorship contribution statement

**Gongchen Zhang:** Methodology, Writing – original draft, Writing – review & editing, Software, Data curation, Validation. **Chenyu Zhu:** Data curation, Validation. **Xiaohan Chen:** Software. **Jifang Yan:** Software, Data curation, Validation. **Dongyu Xue:** Data curation, Validation. **Zixuan Wei:** Data curation, Validation. **Guohui Chuai:** Data curation, Validation. **Qi Liu:** Methodology, Writing – original draft, Writing – review & editing. All authors have read and approved the final manuscript.

## Supplementary material

Supplementary Table S1 **524 candidate BE3 correctable human pathogenic SNP sites**

Supplementary Table S2 **900 candidate ABE7.10 correctable human pathogenic SNP sites**

Supplementary Table S3 **111 candidate human pathogenic SNPs with weak pleiotropic effects (significantly related diseases ≤ 3)**
